# INTEGRATED FAITH BASED CURRICULUM FOR COMMUNITY-BASED OUTREACH WORKERS: ADDRESSING INEQUITIES AMONG OLDER ADULTS

**DOI:** 10.1093/geroni/igac059.3143

**Published:** 2022-12-20

**Authors:** Karen Bullock, Kim Stansbury, Terrance Ruth

**Affiliations:** Boston College, Chestnut Hill, Massachusetts, United States; NC State University School of Social Work, Raleigh, North Carolina, United States; NC State University School of Social Work, Raleigh, North Carolina, United States

## Abstract

Older Latino and Black Americans experience health inequities and a lack of culturally competent clinician may influence this outcome. Community-Based Outreach (CBO) has proven to be effective in addressing such challenges. CBO is an effective approach to engaging communities that have been historically marginalized and are less likely to utilize hospice and palliative than other racial/ethnic groups. Yet, few models and modules for training are available in the literature to prepare CBO workers. This presentation describes the development, coordination, content and implementation of an Interprofessional Educational (IPE) training and curricular innovation to meet needs of diverse older adults diagnosed with a serious illness.

Methods: A community-based outreach approach that integrated faith-based/spiritual care curriculum to train African American and Latino outreach workers to support older adults with serious illnesses, with a focus on advanced care planning (ACP) that explored potential barriers to equitable care. A collaboration with faith-based community representatives to design the intervention research protocol was essential.

Results: The curriculum consisted of three parts: (1) a 36-hour classroom component delivered in three modules organized around eight themes: what is hospice and palliative care, spirituality and the meaning of death, understanding the dying process and decision making, goals of care, culturally responsive care; (2) weekly visits; and (3) field education/workforce development. At the end of the training, learners were able to: (a) express knowledge about culture differences; (b) demonstrate skills – listening, identifying meaning in the decision making, convey health information using patient-centered language; (c) understand cultural influencing decision making.

